# Association between general self-efficacy, social support, cancer-related stress and physical health-related quality of life: a path model study in patients with neuroendocrine tumors

**DOI:** 10.1186/s12955-016-0413-y

**Published:** 2016-01-19

**Authors:** Trude Haugland, Astrid Klopstad Wahl, Dag Hofoss, Holli A. DeVon

**Affiliations:** Diakonova University College, 0130 Oslo, Norway; Faculty of Medicine Institute of Health and Society, University of Oslo, Oslo, Norway; Faculty of Medicine Institute of Nursing Science, University of Oslo, Oslo, Norway; University of Illinois Chicago, College of Nursing, Chicago, IL USA

**Keywords:** General self-efficacy, Social support, Cancer-related stress, Health related quality of life, Interrelationship, Path analysis

## Abstract

**Background:**

A diagnosis of neuroendocrine tumors (NET) provides challenges to patients and clinicians due to physical side effects of and mental response to treatment resulting in increased perceived stress. General self-efficacy, social support and cancer-related stress are key factors in coping. Thus, knowledge of these factors may be of value in improving health-related quality of life (HRQoL). The aim of the study was to examine the relationships between general self-efficacy, social support, cancer-related stress and HRQoL in patients with NET using a path model.

**Methods:**

196 Norwegian patients living with NET were enrolled in this cross-sectional study. Inclusion criteria were: patients with tumors restricted to the GI tract; ability to speak and write Norwegian; over 18 years of age; undergoing medical treatment for NET. Measures used in the study were background characteristics, Health-related Quality of Life (SF-36), the Impact of Event Scale (IES), General Self-efficacy and the Interpersonal Support Evaluation List (ISEL). Relationships between sociodemographic variables, general self-efficacy, social support, cancer-related stress and mental and physical components scores were tested by path analysis with AMOS 22 using maximum standard likelihood estimation.

**Results:**

The sample consisted of 50.5 % women and the average age was 65 years and the median disease duration was 4 years. Sociodemographic variables of gender, education and whether the patient lived alone or with someone were unrelated (directly or indirectly) to HRQoL. Age was directly and negatively correlated with physical HRQoL, general self-efficacy and social support in a well-fitting path model. General self-efficacy modified the negative effects of age on physical HRQoL. Physical and mental HRQoL were not associated with cancer-related stress. Higher social support was related to less stress.

**Conclusion:**

Intervening to improve general self-efficacy and social support for patients with NET may improve their HRQoL.

## Background

Neuroendocrine tumor (NET) is a slow growing cancer and most commonly arises from the gastrointestinal (GI) tract. NET is often advanced at the time of diagnosis and tends to metastasize yet patients may live many years with the disease [1]. NET provides a clinical challenge because it comprises a heterogeneous group of malignancies with a wide range of morphological, functional and behavior characteristics [1]. Tumor burden and symptoms are associated with HRQoL [2] thus, treatment guidelines target symptomatic relief and other strategies to improved quality of life [1]. The physical side effects and the mental response to treatment is often stressful. The experience of stress may include emotional reactions such as intrusive and avoidance thoughts [2]. According to the conceptual model of stress [3] and quality of life [4], stress may be understood as a reaction and symptom of NET and thus, influence a patient’s adaptation to the disease [5–8]. The phenomena of stress and coping and their relationship to health are integral components in caring for chronically ill individuals [3]. Mykletun et al. [9] found that cancer-related stress is strongly related to HRQoL in patients with prostate cancer. However, a longitudinal study found less consistent results suggesting that the two subscales of stress, intrusion and avoidance, associate differently with HRQoL in a variety of cancer diagnosis [10].

General self-efficacy is considered a key factor in coping with NET affecting both adjustment to cancer and HRQoL. General self-efficacy refers to a global confidence in coping abilities across a wide range of demanding situations and reflects a person’s general problem-solving ability [11]. Significant associations between higher general self-efficacy and physical health [12], better mental and physical HRQoL [13], increased physical functioning [14], and increased cancer specific HRQoL [15] have been demonstrated. In patients with NET, higher general self-efficacy was associated with better mental and physical HRQoL [16].

Social support has been shown to play a key role in the coping process [17] enabling individuals to alter the way they view and experience their lives by engaging in a process of cognitive restructuring [18]. Consequently, support from providers may facilitate an individual’s self-regulation by enabling one’s adaptive capabilities to face challenges and to overcome adversity. Higher levels of social support are associated with improved mental HRQoL [12, 13, 15, 19] as well as physical HRQoL [12, 15] in a variety of cancer populations. In addition, patients with NET who have strong social support demonstrated better mental HRQoL than those with less support [16].

Cohen [20] and Haley et al. [21] demonstrated the positive influence of social support and self-efficacy on quality of life. In addition, social support might have beneficial effects on health in times of distress as it buffers the negative impact on stressful events on HRQoL [13, 18, 22]. Kershaw et al demonstrated that self-efficacy and social support are positively related to HRQoL [23]. Furthermore, social support may reduce stress-related arousal and thus provide another source of increased self-efficacy [17]. Modeling relationships between cancer-related stress, general self-efficacy, social support and HRQoL may provide important theoretical and clinical knowledge to enhance the care of patients with NET.

**Fig. 1:**
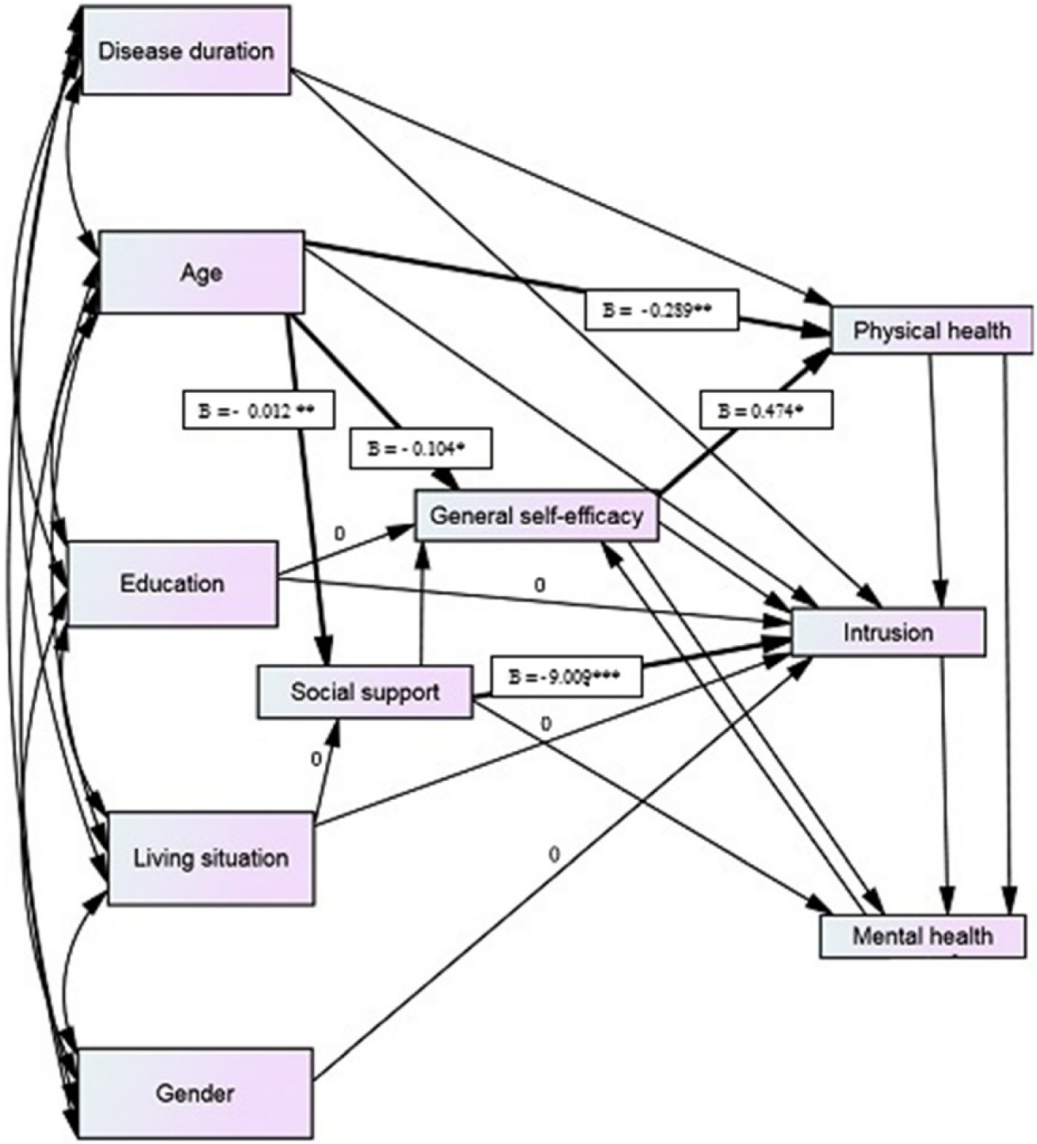
A path diagram of direct and indirect influences of general self-efficacy, social support, cancer-related stress and physical health-related quality of life n mental and physical components of life (*n* = 196). Fit index: χ^2^ = 25.374 Cmin/df = 19, *p* = 0.149, NFI = 0.923, RMSEA = 0.041, Hoelter = 232

In summary, numerous studies have presented the relationship between high levels of stress and poor HRQoL [23], mental HRQoL [13, 22] physical HRQoL [9], physical and social functioning and general health [10] among individuals within a variety of cancer diagnoses. The aim of this study was to test multifactorial path models to evaluate the relationships of general self-efficacy, social support, cancer-related stress and HRQoLin patientws living with NET. Based on the review of the literature, we designed and tested a path analysis model using maximum standard likelihood estimation (Fig. 1).

## Methods

### Design

A cross-sectional design using survey methodology with anonymous, self-reported questionnaires was employed. The Regional Ethics Committee in Health Region II (South) of Norway (S-05156) and the Norwegian Social Science Data Services approved the study (1823) and (2005/AS/PVO-FO-001).

### Sample and procedures

Five NET-centers nationwide provided contact information for 261 patients diagnosed with NET. Inclusion criteria were: patients with tumors restricted to the GI tract; ability to speak and write Norwegian; over 18 years of age; undergoing medical treatment for NET. Patients who were terminally ill, who had undergone radical surgery that may have been curative, or who suffered from cognitive or mental deficiencies were excluded from the study. Patients who had undergone potentially curative surgery were excluded as the aim was to examine general self-efficacy, social support, cancer-related stress and HRQoL in patients living with NET*.* Twenty-five of the 261 patients referred did not meet inclusion criteria. The treatment centers mailed information about the study and questionnaires to the 236 eligible patients. Eligible patients were told that return of a completed survey constituted informed consent. One hundred ninety-six (83 %) (female = 99; male = 97) returned the questionnaire after one reminder.

The survey design precluded us from comparing characteristics of respondents and non-respondents.

### Measures

#### Background characteristics

Gender, age, living alone or with someone, disease duration and level of education were recorded. Level of education was scored as primary, secondary or higher education (college and university). Disease duration was measured in number of years since diagnosis.

#### Health-related quality of life

Health-related quality of life was measured with the SF-36 [24]. The instrument assesses and measures eight dimensions of quality of life. Item scores are linearly transformed into 0–100 scales, and higher scores indicate better HRQoL [24]. The scales and items of the SF-36 have been shown to have satisfactory reliability, validity and responsiveness to changes in health status across patient populations [25], including patients with NET [16]. The eight SF-36 HRQoL subscales were transformed into Physical (PCS) and Mental (MCS) Component Scores, using SF-36 normative data from US populations [24]. A deviation of 10 points from the mean (50) represents a difference of one standard deviation in the general US population [26].

### Cancer-related stress

The Impact of Event Scale (IES) [2] was modified and used to measure current subjective stress related to cancer. The words “life events” were changed to “cancer” so that participants would respond to questions from the perspective of their cancer diagnosis. Seven items assess intrusive thoughts, which can be described as invasive ideas, images, feelings or bad dreams. Eight items assess avoidance thoughts that are described as conscious evasion of certain ideas, feelings or situations for events related to cancer. Each item is scored on a 6-point scale from 0 (never) to 5 (often). The total score for all 15 items ranges from 0 to 75, with higher scores indicating higher disease-related stress. Impact of Event Scale scores of 8 or below indicate “low” stress, scores ranging from 9 to 43 represent “mild to moderate” stress, and scores 44 or higher represent severe stress [2]. The IES questionnaire has been found to be a valid and reliable measure of stress in patients with NET [16].

### General self-efficacy

The General Perceived Self-efficacy Scale is a 10-item scale that measures general self-efficacy. Each item is scored from 1 (quite wrong) to 4 (quite right). Summary scores range from 10 to 40; higher scores indicate more optimistic self-beliefs. The scale has demonstrated satisfactory validity and reliability in patients with NET [16].

### Social support

The Interpersonal Support Evaluation List (ISEL) is comprised of 30 statements designed to assess the perceived availability of five types of social resources [5]. The five scales measure: Appraisal Support; Self-esteem Support; Group Belonging; Emotional Closeness Support, and Tangible Support. Each scale consists of six statements that are worded both positively and negatively, and are measured on a 4-point scale that ranges from 1 (definitely true) to 4 (definitely false). Higher scores indicate higher perceived support [5]. The five scales were summed to determine an overall score.

### Statistical analyses

Data were analyzed with SPSS version 22. The level of statistical significance (two-sided) was set at <0.05 for all analyses. Descriptive statistics were performed to assess the background characteristics **(**gender, age, living with partner or not, and level of education) of the sample (Table 1). Missing scores in the SF-36 questionnaire were handled in accordance with the SF-36 manual [24] in that, mean substitution was used to calculate the score for dimensions when fewer than 50 % of the scores were missing.

**Table 1:** Patients characteristics (*n* = 196)

Gender	
Male n (%)	97 (49.5)
Female n (%)	99 (50.5)
(missing = 7)	
Age mean years (SD, range)	65 (11, 33–85)
Living with partner or not (*n*, %)	
Divorced, widowed, living with children	49 (25)
Married and/or cohabitation	147 (75)
Education n (%)	
Primary	72 (41)
Secondary	47 (27)
College/University	43 (24)
(missing = 20)	14 (8)
Mean disease duration in years (SD, median, range)	4.8 (4.3, 4, 0–23)
(missing = 15)	

*Abbreviations: SD* standard deviation

The relationship between general self-efficacy, social support, cancer-related stress and mental and physical components scores was tested using path analysis with AMOS using maximum standard likelihood estimation. Model fit was evaluated by; goodness-of-fit indices; model chi-square value, *p* value; Cmin/df; the Normed Fit Index (NFI); the Root Mean Square Error of Approximation (RMSEA) , and the Hoelter 05 [27]. The original model, derived from existing research, was first tested for acceptability and improvement possibilities on a random 50 % of the sample. The adjusted model was tested and confirmed in the total sample. To maintain comparability of models through the model adjustment process, unimportant variables were not removed from the model, but non-significant relationships *p* > 0.05 between variables were constrained to zero.

## Results

The sample consisted of 50.5 % women. The average age of patients was 65 years and the median disease duration was 4 years. (Table 1). The average physical HRQoL score was 39.6, a lower score than the norm referenced population (mean = 50). The mean stress score for the IES (Avoidance & Intrusion Scales) was 24, indicating that the majority of patients experienced mild to moderate stress. Twelve percent of the sample reported a severe stress response (score ≥ 44). The mean score for general self-efficacy was 30, indicating a low level of self-confidence. The mean score for ISEL (social support) was 3.1 indicating high levels of social support among the majority of the patients (Table 2).

**Table 2:** Mean values, SD and range of, General Self-efficacy Scale, Interpersonal Support Evaluation List, Impact of Event Scale and SF-36 physical and mental HRQoL (*n* = 196)

Variable	Mean (SD, range)	*N* (%)
General Self-efficacy Scale	29.9 (5.5, 10–40)	
Low (≤30)		109 (55.6)
High (≥ 30)		83 (42.3)
(missing = 4)		
Interpersonal Support Evaluation List	3.1 (0.5, 1.7–3.9)	
Low (≤ 2)		4 (3.6)
High (≥ 2)		112 (55)
(missing = 84)		(42.9)
Impact of Event Scale	24 (16, 0–74)	
Mild ≤ 8		37 (18.9)
Moderate 9–43		130 (66.3)
(missing = 5)		
SF-36		
Physical HRQoL (missing = 15)	39.6 (11, 10–59)	
Mental HRQoL (missing = 15)	45.9 (11, 15–66)	

*Abbreviations: SD* standard deviation, *HRQoL* health related quality of life

### The relationships between general self-efficacy, social support, cancer-related stress and health-related quality of life

We tested the model by analyzing the total scores of IES as well as the two subscales of intrusive thoughts and avoidance. The best model fit was achieved by using the intrusive subscale. Thus, the model analyzing the intrusive subscale was used. Performing path analysis produces two types of results; estimates of the model’s fit to the data and, estimates of the strength of the relationships between the variables in the model. The tested model was a very good fit (χ^2^ = 25.374 (df = 19), *p* = .149, Cmin/df = 19, NFI = 0.923, RMSEA = 0.041 and, Hoelter = 232) to the total data set after some adjustments based on a randomly selected 50 % of cases (Fig. 1). The relationships between age and physical HRQOL (β = −0.289), self-efficacy (β = −0.104), and social support (β = −0.012) were significant (*p* < .05) by standardized coefficient estimates for the paths. Self-efficacy was positively related to physical HRQOL (β = 0.016). The standardized coefficient estimate between social support (β = 0.04) and cancer-related stress as measured by intrusive thoughts was also significant. The model demonstrated that cancer-related stress was not significantly associated with physical or mental HRQoL. The socio-demographic background variables were weakly related to HRQoL and the other variables in the model. Finally, the model fit was not weakened by constraining these coefficients to zero.

## Discussion

This study aimed to test a multifactorial path model (Fig. 1) to evaluate the relationship between general self-efficacy, social support, cancer-related stress and HRQoL in patients diagnosed with NET. The key findings of this study were that age was directly and negatively correlated with physical HRQoL, self-efficacy and social support. Self-efficacy mediated the relationship between age and physical HRQoL demonstrating that older persons with less confidence in their self-efficacy had lower levels of physical HRQoL.

The symptoms of NET are related to site of the tumor and aggressiveness, the degree of metastases, tumor burden, reduced physical functioning and time since diagnosis [16]. Symptoms also vary by treatment, severity and aggressiveness of the cancer [1] The mean disease duration for the NETs was 5 years, however we could not determine the aggressiveness of the disease. Vinik et al. [2] found no association between aggressiveness and any of the quality of life domains for NET patients. As many of the patients in the present study have had the diagnosis for more than 5 years, it is possible that some patients had developed a more advanced disease while others had a more favorable disease course.

Our findings partially support prior research demonstrating that social support and self-efficacy influence quality of life [20, 21]. Our results did not demonstrate a direct relationship between social support and physical HRQoL but showed a direct positive relationship between social support and cancer-related stress (intrusive thoughts). Our results suggest that social support may mitigate the influence of stressful events on health in times of distress. This differs from previous research [9, 13, 18, 22, 23], in which a direct relationship between cancer-related stress and HRQoL was established. These inconsistent results may reflect the construction of the IES, heterogeneity of the sample or variation in statistical methods. It has been argued [3], that stress should not be measured as a single construct. Stress may be understood as an umbrella term for a complex series of subjective phenomena, including cognition, appraisals, stress emotions, coping response and reappraisals. Stress is experienced when the demands of a situation tax or exceed a person’s resources and harm or loss is anticipated [3]. Therefore, cancer-related stress may not be fully captured by the IES. Still, our results confirm the idea that promoting social support among older patients whose natural network may be diminishing may reduce levels of cancer-related stress by buffering intrusive thoughts. Like previous research, [13–16] our analysis confirmed that physical HRQoL is related to general self-efficacy.

In contrast to prior studies, we found no association between cancer-related stress and physical or mental HRQoL. A previous literature review [28] concluded that for women diagnosed with breast cancer, aging was associated with greater psychological stress. A study of a large sample of prostate cancer survivors 5 years after diagnoses demonstrated that cancer-related stress was strongly associated with HRQoL [9]. One explanation could be that our NET patients were older (mean age 65 years) than the prostate cancer survivors (mean age 41 years at diagnosis). However; based on the literature review, age was not considered to be negatively associated with social support. In contrast to the literature, our path model showed that social support had a direct relationship with intrusion thoughts. The results suggest that NET-patients with more social support experienced less intrusive thoughts than those with less support and that social support mediated age in the relationship with intrusive thoughts. A good social network may therefore be a possible explanation for the different results. If this is so, interventions aimed at increasing the NET patients’ social network and motivating them to take part in social events may be an option to reduce the experience of cancer-related stress.

### Strengths and limitations

Strengths of our study included sampling and response rate. Nearly the entire population of NET patients in Norway were included in the study and our response rate was 83 %. Our analyses are also strength. Path analysis is methodologically superior to other statistical models, such as multiple regression, because it shows the presence and magnitude of direct and indirect relationships. The cross-sectional design of our study was a limitation. We were unable to identify the trajectories of self-efficacy, social support and physical HRQoL which may change over the course of NET cancer. We were also unable to measure the stage of the disease or the individual’s knowledge of their own prognoses which could have influenced their level of self-confidence and may have confounded results.

Our results are congruent with previous studies of individuals at risk of hereditary cancer [13] and infected with HIV which demonstrated that higher levels of general self-efficacy were associated with better physical health and increased physical functioning [14].

Clinical implications include focusing efforts on improving self-efficacy in patients with NET through counseling and education.

## Conclusion

Sociodemographic variables of gender, education and whether the patient lived alone or with someone were unrelated (directly or indirectly) to HRQoL. Age was directly and negatively correlated with physical HRQoL, general self-efficacy and social support in a well-fitting path model. Self-efficacy mediated the relationship between age and physical HRQoL demonstrating that older persons with lower confidence in their self-efficacy had poorer physical HRQoL. There was no direct association of age on cancer-related stress (intrusive thoughts) however, older persons with less social support experienced more intrusive thoughts. Thus, focusing on strengthening the NET patients’ general self-efficacy and social support may positively influence physical HRQoL.

## Abbreviations

GI: gastrointestinal
HRQoL: health related quality of life
IES: the impact of event scale
ISEL: the interpersonal support evaluation list
MCS: mental component scores
NET: neuroendocrine tumor
PCS: physical component scores
SD: standard deviation
